# Multi-omics and AI-driven advances in miRNA-mediated hair follicle regulation in cashmere goats

**DOI:** 10.3389/fvets.2025.1635202

**Published:** 2025-07-09

**Authors:** Zhang Chunhua, Fu Le, Li Shengli, Wu Sachula, Hua Bao, Mu Lan, Marco Antonini, Sun Haizhou

**Affiliations:** ^1^Institute of Animal Nutrition and Feed, Academy of Agriculture and Animal Husbandry Sciences, Hohhot, China; ^2^Key Laboratory of Grass-Feeding Livestock Healthy Breeding and Livestock Product Quality Control (Co-Construction by Ministry and Province), Ministry of Agriculture and Rural Affairs, Hohhot, China; ^3^Inner Mongolia Key Laboratory of Herbivore Nutrition Science, Hohhot, China; ^4^ENEA Casaccia Department BAS, Secondment at the University of Camerino Industrial Liaison Office, Rome, Italy

**Keywords:** velvet goat, hair follicle, miRNA, cashmere, AI-powered multi-omics

## Abstract

Hair follicle development and cycling are governed by intricate genetic and molecular networks, with microRNAs (miRNAs) playing essential roles as post-transcriptional regulators. In cashmere goats, valued for their fine fiber, miRNAs have emerged as key modulators influencing hair follicle morphogenesis, regeneration, and fiber traits such as fineness and pigmentation. This review highlights recent discoveries in miRNA-mediated regulation of hair follicles, focusing on their dynamic expression patterns and cell-specific functions in keratinocytes, dermal papilla cells, and follicular stem cells. Key miRNAs, including miR-31, miR-22, and miR-214, are explored for their effects on follicle growth, hair shaft formation, and pigment regulation. We discuss advances in single-cell RNA sequencing and spatial transcriptomics, revealing new insights into cellular heterogeneity and lineage specification. Integrative multi-omics approaches, combining transcriptomics, proteomics, and epigenomics uncover complex regulatory networks in which miRNAs interact with other non-coding RNAs and signaling pathways. Artificial Intelligence (AI) -driven analytics enhance the discovery of biomarkers and therapeutic targets, offering precision strategies for clinical and livestock applications. miRNA profiling now informs breeding strategies to improve cashmere fiber quality and is a minimally invasive diagnostic tool for hair disorders. We outline future directions, including improved miRNA delivery methods, systems biology integration, and AI-powered multi-omics approaches to deepen our understanding of hair follicle biology and facilitate practical applications in medicine and agriculture.

## 1 Introduction

MicroRNA (miRNA) is a small non-coding RNA molecule, approximately 22 nucleotides in length. It regulates gene expression post-transcriptionally, influencing cell proliferation, cycle progression, epigenetics, and apoptosis (1). Importantly, miRNAs are involved in the regulation of mammalian hair growth (2). The exploration of miRNA-mediated regulation in hair follicles holds broader implications beyond basic science (3). Cashmere, derived from the soft undercoat of *Capra hircus*, ranks among the world’s most valuable animal fibers, with a luxury market worth USD 2.8 billion in 2023 and growing at a 6.2% CAGR through 2030 (4). Global raw cashmere production ranges from 13,000–18,000 tons annually, with China and Mongolia contributing over 90% (5, 6). Individual goats produce just 150 g per year, translating to approximately $10 USD in market value per goat for rural herders (7). The fiber’s softness, insulating properties, and fine diameter (<19 μm) make it highly sought after in luxury textiles (8). Understanding the molecular mechanisms, particularly miRNA-mediated regulation, that govern fiber development is therefore critical for improving production efficiency, breeding precision, and fiber quality (9). These insights directly support both economic sustainability and genetic innovation in high-value livestock systems. Likewise, in human hair disorders such as androgenetic alopecia and alopecia areata, deregulated miRNAs (e.g., miR-125b, miR-22, miR-214) have been implicated in hair loss pathogenesis (10, 11). Understanding these pathways not only advances diagnostic biomarker discovery but also supports the development of miRNA-based therapeutic strategies for clinical dermatology (12). Understanding these regulatory mechanisms holds significant implications not only for improving cashmere fiber traits in livestock breeding but also for advancing diagnostic and therapeutic strategies for human hair disorders such as alopecia and scalp inflammation (13). For instance, miRNA-203 was the first to be identified as abundantly expressed in the epidermis and hair follicles (14). This suggests a strong link between miRNAs and hair follicle development. An *in vitro* study conducted on a mouse has demonstrated that miR-200b and miR-196a play critical roles in hair follicle development through interactions with target genes in the WNT signaling pathway (11).

Comparative studies across mammalian species have revealed both conserved and species-specific roles of miRNAs in hair follicle regulation (15, 16). For example, miR-125b, known for regulating epidermal differentiation and sebaceous gland size in mice, also inhibits hair shaft formation and causes fur growth defects when overexpressed (16). In human scalp tissue, miR-22 and miR-31 are differentially expressed in androgenetic alopecia, modulating keratinocyte proliferation and hair cycle phase transitions (17). In sheep, miRNAs such as miR-143 and miR-200b have been linked to wool pattern and follicle morphology, influencing FGF and Wnt signaling (18). Meanwhile, in cashmere goats, miRNAs including miR-214, miR-29a/b1, and miR-199a-5p target pathways like WNT, BMP, and TGF-*β*, directly affecting hair follicle stem cell activity, coat color, and fiber fineness (19). These cross-species comparisons demonstrate evolutionary conservation in key signaling axes while highlighting the unique regulatory networks that underlie cashmere-specific traits, supporting a focused investigation of miRNA functions in this economically important breed.

Studies on various sheep and goat breeds, including Liaoning, Inner Mongolia, and Shaanbei white cashmere goats, have also revealed significant miRNA expression changes during hair follicle development, underscoring their regulatory roles in hair growth (20). A longitudinal study spanning 12 months on the skin tissue of Albas white cashmere goats in Inner Mongolia highlighted that the secondary hair follicle cycle could be divided into a growth phase from March to September, a resting phase from September to December, and a regression phase from December to March (21). Keratin gene expression followed the hair follicle’s seasonal growth cycle. These findings support further genetic studies on hair follicle cycling. miRNAs are pivotal in modulating hair follicle development and morphogenesis in velvet goats (22). These small, non-coding RNAs are integral to ensuring the correct formation and spatial organization of hair follicles, which collectively influence the phenotypic characteristics of the velvet goat’s hair coat.

Single-cell RNA sequencing (scRNA-seq) enables detailed analysis of the cellular composition and developmental processes of hair follicle (23). Using UMAP, researchers identified 19 cell populations from 15,830 single-cell transcriptomes and mapped their identities via gene expression profiles. A novel marker gene was employed to identify *in vitro* isolated dermal papilla cells (24). Pseudo time analysis revealed the differentiation trajectory of matrix progenitors into hair shaft and inner root sheath (IRS) cells. Differential gene expression analysis between straight and curly wool identified potential molecular mechanisms underlying wool curvature (25).

Androgenetic alopecia (AGA), a prevalent form of hair loss that affects both men and women, is primarily driven by a genetic predisposition and enhanced sensitivity to androgens. To better understand its pathophysiology and develop therapies, researchers increasingly rely on multi-omics technologies (26). This interdisciplinary approach integrates a range of techniques, including genomics, transcriptomics, proteomics, and metabolomics, which collectively provide profound insights into both basic and clinical medicine (27). Numerous studies have reported significant alterations in miRNA, proteins, and metabolites in affected individuals, thereby advancing our comprehension of underlying mechanisms. The application of multi-omics technologies facilitates a more integrated and comprehensive understanding of the molecular landscape, enabling the identification of potential therapeutic targets and the development of personalized treatment strategies (28). In particular, the incorporation of artificial intelligence (AI) and single-cell multi-omics approaches has significantly transformed the field of hair follicle research. These tools enable precise profiling of cell types and discovery of novel biomarkers, such as miRNAs, that play crucial roles in pathogenesis by enabling a more granular investigation of the molecular events occurring in hair follicles (29). This review aims to (i) synthesize current insights into miRNA-mediated regulation of hair follicle development in cashmere goats; (ii) explore cell-type specific roles of miRNAs; (iii) examine technological advances like single-cell sequencing and AI in miRNA research; and (iv) assess their translational potential in diagnostics, therapeutics, and livestock breeding strategies to revolutionize our understanding and treatment strategies while also addressing the challenges and future directions in this rapidly evolving field.

## 2 Hair follicle development and growth cycle

### 2.1 Structure and morphogenesis

**Figure 1:**
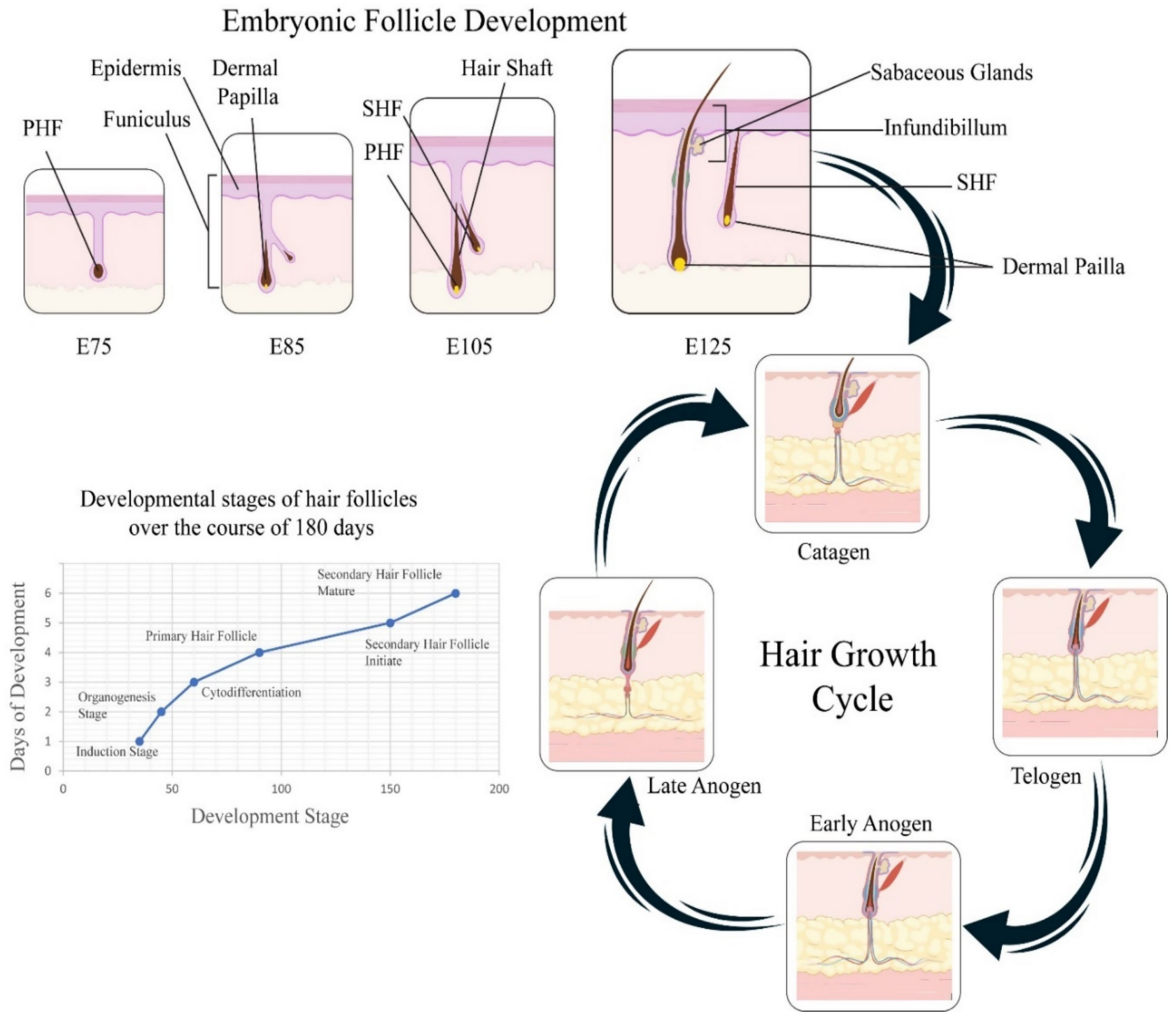
Embryonic and postnatal hair follicle development in cashmere goats. The top panel shows sequential embryonic stages of primary (PHF) and secondary (SHF) hair follicle development from E75 to E125, with key structures such as the dermal papilla, epidermis, and funiculus labeled. The lower left graph summarizes developmental stages over 180 days, progressing from follicle induction to maturation. The right panel depicts the cyclic hair growth phases, early and late anagen (growth), catagen (regression), and telogen (rest), highlighting the morphological changes of hair follicles across these stages.

Hair follicles, as accessory organs of the skin, serve as the fundamental units for the formation of villi within the dermis. Their development is initiated during the embryonic stage (30). Morphologically and temporally, the progression of hair follicle growth is divided into three critical phases: the induction phase (embryonic days 55–65), the organogenesis phase (embryonic days 85–95), and the cell differentiation phase (embryonic days 115–125) (30). Mature secondary hair follicles are structurally categorized into the infundibulum, isthmus, and lower follicle segments comprising dermal papilla cells, hair matrix cells, inner root sheath cells, and outer root sheath cells (31). Owing to the coordinated biological regulation by these cells, the development of secondary hair follicles is cyclical, undergoing self-renewal across three stages: anagen (growth), catagen (regression), and telogen (rest) (32). Hair follicles, although they vary in size and morphology based on anatomical location, maintain a consistent internal structure composed of basic components. At the base of the follicle resides the hair bulb, where the hair shaft is generated by proliferating matrix keratinocytes. Between these cells are melanocytes, which synthesize melanin to provide pigment to the developing hair (33). As differentiation progresses, the growing hair shaft rises, forming a mature fiber composed of intermediate filaments and structural proteins that constitute the cortex. The dermal papilla, a cluster of specialized mesenchymal cells located beneath the matrix zone, plays a central role in controlling hair production by controlling matrix cell proliferation and, consequently, determining the size of the hair shaft (33). In cashmere goats, primary hair follicles (PHFs) begin forming around embryonic day 65, followed by secondary hair follicles (SHFs) initiating between days 85 and 105, with full maturation typically observed by E125 (34). This timeline corresponds with morphological changes depicted in the upper panel of Figure 1.

In the early stages of follicular development, specific miRNAs regulate the expression of genes controlling the proliferation, differentiation, and spatial patterning of follicle stem and progenitor cells (35). These miRNAs target transcription factors and signaling pathways crucial for the initiation and subsequent morphogenesis of hair follicles. As hair follicles advance through developmental phases, miRNAs maintain their regulatory roles by influencing gene expression involved in the growth, cycling, and differentiation of follicular cells (36). For instance, they regulate the expression of genes vital for the development of the hair shaft, the inner and outer root sheaths, and the dermal papilla, all of which are integral to the hair follicle’s architecture (37). The fine-tuned regulation of miRNA expression during hair follicle morphogenesis in velvet goats is achieved through sophisticated mechanisms such as post-transcriptional control of miRNA biogenesis, epigenetic modifications, and intricate feedback loops (38). These processes ensure precise spatiotemporal expression patterns of miRNAs, which are critical for controlling gene expression programs that dictate the development of hair follicles and, ultimately, the physical traits of the velvet goat’s coat (39).

### 2.2 Hair follicle cycling: anagen, catagen, telogen

Hair growth occurs through a cyclic process comprising four distinct phases: anagen, catagen, telogen, and exogen. These phases occur asynchronously across individual follicles, with each follicle undergoing between 10 to 30 cycles over the human lifespan (40, 41). This asynchronous cycling contributes to the maintenance of consistent hair density under normal physiological conditions. The anagen phase is the most prolonged and metabolically active stage of the hair cycle, characterized by rapid cellular proliferation and elongation of the hair shaft (42). During this phase, the follicle is deeply embedded in the dermis, and the complete hair fiber is formed (42). On the scalp, the anagen phase typically lasts between two to 8 years, enabling significant hair growth. In contrast, follicles in regions such as the eyebrows exhibit a much shorter anagen phase of only two to 3 months, resulting in limited hair length (43). Hair shaft length, in the absence of mechanical trimming, directly reflects the duration of the anagen phase. However, with advancing age, the duration of anagen shortens, and the proportion of follicles remaining in this phase decreases, leading to progressively thinner and weaker hair (44). Anagen hair shedding, known as anagen effluvium, occurs when this growth phase is prematurely terminated or arrested due to external factors such as chemotherapy or radiation. Unlike telogen shedding, anagen effluvium is considered pathological (45).

Following anagen, the follicle enters the catagen phase, a brief transitional period lasting approximately 2 weeks. This phase marks the regression of the follicle, during which mitotic activity in the matrix ceases, and programmed apoptosis is initiated in the epithelial compartment of the bulb (46). The hair follicle detaches from the dermal papilla, which subsequently migrates upward toward the follicular bulge. The successful reconnection of the dermal papilla with the bulge region is essential for the re-initiation of follicular cycling. If this interaction fails, follicular cycling halts permanently, often resulting in irreversible hair loss (47). The catagen phase is succeeded by the telogen phase, a resting stage that typically spans two to 3 months. During telogen, the follicle remains quiescent, and no new hair is actively produced (48). Approximately 9% of scalp follicles reside in telogen at any given time, compared to a higher percentage around 40–50% in body hair follicles, such as those on the trunk. While the older hair remains in place, a new anagen hair begins to form beneath it, slowly pushing it toward the surface (49). Disruptions that cause follicles to prematurely enter telogen can lead to a condition known as telogen effluvium (TE), characterized by diffuse and excessive hair shedding. This condition may be triggered by systemic stressors, hormonal imbalances, or nutritional deficiencies (50). Conversely, therapeutic approaches that reduce the proportion of follicles in telogen have demonstrated benefits in mitigating hair loss and promoting regrowth. The final stage, exogen, represents the culmination of the telogen phase and the initiation of a new anagen cycle (40). During exogen, the newly formed hair shaft continues to ascend, ultimately displacing and shedding the telogen hair fiber from the follicle. This shedding is a physiologically normal event and essential for the renewal of the hair cycle (51). On average, healthy individuals shed approximately 100 to 150 hairs daily, a number that reflects the proportion of follicles naturally residing in telogen and entering exogen. Because follicular cycling is asynchronous, overall hair density remains relatively stable in the absence of pathological disruptions (52).

The hair follicle cycle in cashmere goats is uniquely adapted to seasonal rhythms, with a well-defined annual cycle: anagen from March to September, catagen from October to December, and telogen from January to February, tightly linked to photoperiod and hormonal cues (45). In contrast, mice exhibit much shorter follicle cycles (weeks) that are synchronized across the skin, while humans undergo asynchronous follicle cycling with individual follicles independently transitioning through phases (53). Additionally, sheep breeds such as Merino often exhibit longer anagen phases to support wool growth, but with less pronounced seasonal shifts compared to cashmere goats (54). These interspecies differences underscore the species- and breed-specific miRNA regulatory programs controlling hair growth, making the cashmere goat a unique model for studying seasonal follicle dynamics and fiber specialization.

Numerous miRNAs exhibit phase-specific expression during the hair follicle cycle, influencing distinct biological events. For instance, miR-31 is highly expressed during the anagen phase, where it promotes hair shaft elongation and keratinocyte proliferation by targeting genes such as DLX3, FGF10, and various keratins (55). Conversely, miR-22 is upregulated during the catagen and telogen phases, where it represses keratinocyte differentiation and accelerates follicular regression by targeting transcription factors like DLX3, HOXC13, and Foxn1 (56). Additionally, miR-125b, enriched in hair follicle stem cells (HFSCs), is known to maintain stemness during telogen and prevent premature activation of follicle cycling, contributing to follicle quiescence (57). These examples demonstrate that miRNAs not only fluctuate across the hair cycle but also play active roles in coordinating transitions between phases through modulation of proliferation, differentiation, and apoptosis.

Understanding the dynamics of the hair follicle cycle is of practical importance for improving cashmere production. Since cashmere fibers are derived from secondary hair follicles, which follow a seasonal growth cycle, precise knowledge of anagen timing and duration directly influences fiber length and yield (58). Early or prolonged anagen phases result in longer, finer fibers, while premature entry into catagen or telogen can reduce fiber quality (40). By identifying miRNAs and signaling pathways that regulate follicle cycling, especially those tied to phase transitions, researchers can develop molecular tools for selective breeding and optimize environmental management practices (e.g., photoperiod manipulation) to enhance fiber traits in cashmere goats (59).

As illustrated in Figure 1, the development of cashmere goat hair follicles involves two main temporal domains: embryonic morphogenesis and postnatal cyclic regeneration. The top panel depicts the sequential formation of primary hair follicles (PHFs) around embryonic day 65 and secondary hair follicles (SHFs) between E85 and E125 (60). These structures show increasing morphological complexity, with the appearance of the dermal papilla, funiculus, and sebaceous glands as development proceeds. The bottom-left graph visualizes the progression of follicular stages, induction, organogenesis, cytodifferentiation, and maturation, over approximately 180 days, corresponding with both cellular and molecular transitions that guide the formation of mature follicles. The right panel outlines the cyclic phases of the hair growth cycle postnatally: early and late anagen (growth), catagen (regression), and telogen (rest). This panel illustrates key morphological changes, such as follicle elongation, shrinkage, and quiescence, emphasizing the seasonal control of fiber production in cashmere goats. Collectively, Figure 1 integrates spatial and temporal elements of hair follicle development, offering a holistic visualization of folliculogenesis and cycling.

### 2.3 Key regulatory genes and pathways

Hair follicle development and cycling are orchestrated by a complex network of molecular signaling pathways, in which the canonical WNT and BMP pathways play crucial roles. Additionally, miRNA have been implicated in modulating the morphogenesis and regeneration of hair follicles (52). These diverse signaling cascades of regulatory factors integrate an intricate molecular framework that governs hair follicles’ proper morphogenesis and cycling regeneration. Precise regulation of these signaling networks is critical for normal follicle development and hair cycle. Dysregulation of these pathways can result in various hair follicle-related disorders (61). Key signaling pathways implicated in hair follicle morphogenesis include WNT, BMP, EDAR, Notch, and Sonic Hedgehog. Aberrations in ligands, receptors, and intracellular transduction components of these pathways can disrupt hair follicle development, ultimately affecting hair growth dynamics (62). Certain genes have been identified as positive regulators of early hair follicle morphogenesis, include WNT/*β*-catenin, WNT10b, LEF1, and EDAR in the epidermis, as well as WNT/β-catenin, WNT5a, LEF1, and Noggin in the dermis. Conversely, genes known to inhibit hair follicle morphogenesis include DKK4 and BMP2 in the dermis, and DKK1, BMP4, and BMP7 in the dermis (63, 64).

The WNT/*β*-catenin signaling pathway is a critical regulator of hair follicle morphogenesis and cycling, initiating follicle development through basal plate formation. Canonical WNT signaling involves WNT ligands, Frizzled receptors, Disheveled (DSH) proteins, β-catenin, and the axin/GSK3/APC complex (65). WNT proteins, secreted via Wntless (Wls), are classified into primary (WNT3, WNT4, WNT6) and secondary (WNT2, WNT7b, WNT10a, WNT10b) subgroups. Primary WNTs induce follicle formation, while secondary WNTs promote development (66). WNT3a is predominantly expressed in progenitor cells, bulge, and melanocytes, contributing to melanocyte proliferation and pigmentation. WNT10b is highly expressed during anagen, promoting epithelial differentiation and inducing transition from telogen to anagen. TNF-*α* enhances WNT3 and WNT10b expression via NF- κB activation. *β*-catenin expressed in ORS, IRS, matrix, and dermal papilla cells (DPCs), activates LEF/TCF complexes in hair follicle stem cells (HFSCs), inducing downstream genes like *c-myc* and *cyclin D1*, regulating proliferation and differentiation (67). Long non-coding RNA H19 sustains follicle inductivity by repressing WNT inhibitors like *DKK1*, *Kremen2,* and *SFRP2,* while upregulating *miR-29a*. Blimp1 also act as both target and effector of WNT/ *β*-catenin and TGF-β pathways in DPCs. This integrated signaling network is essential for the initiation, growth, and regeneration of hair follicles (68, 69).

The BMP (Bone Morphogenetic Protein) signaling pathway is another essential regulator of hair follicle morphogenesis and cycling, primarily acting as an inhibitory signal for hair growth (70). BMPs are secreted glycoproteins within the TGF-*β* β superfamily that exert their function by binding to serine/threonine kinase BMP receptors, forming an active heterotetrameric complex. This complex phosphorylates Smad1/5/8, which then associates with Smad4 andtranslocates to the nucleus to initiate transcription of genes involved in the regulation of hair follicle stem cells (HFSCs) proliferation and differentiation (70). Inhibitory Smad5, Smad6, Smad7, and Smad7 antagonize BMP signaling by competing with Smad4 or promoting Smad1/5/8 degradation. BMP signaling is dynamically active during the telogen phase, modulating the transition between the refractory and regenerative phases (71). BMP activity suppresses hair follicle base plate formation, while its antagonist Noggin enhances follicle induction and shortens the refractory period, promoting hair regeneration. Furthermore, during wound healing, myofibroblasts can convert into adipocytes, a process dependent on BMP signaling that activates adipocyte transcription factors (63, 72). BMP signaling alsoth contributes to determining eccrine sweat gland fate during development, and knockout of BMPR1A leads to sweat gland cells adopting hair follicle-like characteristics. Thus, BMP signaling functions as a context-dependent modulator of skin appendage fate and follicle activity (73).

The EDAR signaling pathway plays a crucial role in hair follicle development and cycling. It comprises the ectodysplasin A (EDA) ligand, its transmembrane receptor EDAR (including EDA-A1 and EDA-A2 isoforms) and the intracellular adapter protein EDARADD. Both EDA and EDAR belong to the tumor necrosis factor (TNF) superfamily (63, 74, 75). Structurally, EDAR possesses an extracellular ligand-binding N-terminal domain, a single transmembrane region, and an intracellular death domain that binds specifically to EDARADD, initiating downstream signaling cascades and transcription of target genes (76). During the hair follicle cycle in wild-type mice, EDA, EDAR, and EDAEADD show dynamic expression patterns, peaking at the end of anagen, declining through catagen, and reaching their lowest levels in telogen (76). EDA-A1 is expressed in the hair matrix, inner root sheath (IRS), and outer root sheath (ORS) during late anagen, with diminished expression limited to secondary follicle buds in mid-catagen. EDAR expression rises in the IRS and ORS toward the end of anagen and localizes to secondary follicle buds by late catagen. This stage-specific and compartmentalized activity of the EDAR pathway underscores its key role in regulating hair follicle morphogenesis and regeneration (77).

Gene expression profiling has revealed dynamic and pathway-specific miRNA regulation during hair follicle development. For instance, miR-214, a known inhibitor of the WNT/*β*-catenin pathway, shows peak expression during telogen, where it represses β-catenin and downstream targets such as c-Myc and cyclin D1, thereby inhibiting follicle activation (78). Conversely, miR-22, which regulates the TGF-β and BMP pathways, is upregulated during catagen, where it promotes regression by targeting SMAD6 and keratinocyte-related transcription factors like DLX3 and Foxn1 (11). In contrast, miR-29a/b1 targets LRP6 and Bmpr1a, suppressing both WNT and BMP signaling, and is differentially expressed across the hair cycle, typically upregulated in late anagen or early catagen (79). These expression patterns underline the role of miRNAs as phase-specific fine-tuners of signaling cascades that control follicle morphogenesis, growth, and quiescence.

In cashmere goats, recent transcriptome and miRNA sequencing studies have validated these interactions. For instance, miR-214 was shown to suppress *β*-catenin expression in dermal papilla cells of cashmere goats, leading to decreased proliferation during telogen, mirroring its inhibitory role observed in murine models (80, 81). Similarly, miR-29a/b1 targets Bmpr1a and LRP6, disrupting BMP and WNT signaling and correlating with lower follicle density and fiber thickness in low-yielding goat breeds (79). These findings confirm that miRNA-pathway interactions are not only conserved but also species-specific in their phenotypic outcomes, providing critical insights for breeding strategies in cashmere goats.

## 3 miRNAs in cashmere goat hair follicle development

### 3.1 Identification and expression patterns

Micro RNAs regulate gene expression in many species, including velvet goats, where they control hair follicle morphogenesis and development. These miRNAs influence cell proliferation, differentiation, and apoptosis, affecting hair characteristics. Studies estimate that miRNAs regulate up to 30% of protein-encoding genes, underlining their importance in hair follicle structure and maintenance (82). Recent studies reveal that miRNAs significantly impact biological pathways by regulating associated genes (83). In Jiangnan cashmere goats, 193 differentially expressed miRNAs (DE miRNAs) were identified across the hair follicle cycle, showing 1,472 regulatory relationships with target genes. These miRNAs include chi-miR-17-5p and chi-miR-199a-5p, which are vital for regulating key genes such as BAMBI and SMAD1 (21). Additionally, research on Inner Mongolia and Liaoning cashmere goats found a complex regulatory network involving differentially expressed mRNAs, lncRNAs, and circRNAs, contributing to the fineness of their hair (84).

The miRNA regulatory network is highly intricate and dynamic, with miRNAs regulating one another and influencing multiple target genes and non-coding RNAs. Additionally, miRNAs can act as “sponges” by binding to target gene sequences, preventing miRNAs from interacting with their natural targets, thereby inhibiting target gene expression (85). For example, lncRNA H19 enhances the viability and proliferation of dermal papilla cells in cashmere goats, but chi-miR-214-3p binds to the *β*-catenin 3′ UTR, blocking H19’s effect and suppressing dermal papilla cell proliferation (86, 87). A study identified multiple miRNAs enriched in the Notch and TGF-β pathways, illustrating their influence on secondary hair follicle development. miRNA-21, a downstream component of BMP signaling, negatively regulates genes such as CNKSR2, KLF3, and TNPO1 during hair follicle development (88). In Cashmere Goats, miRNAs significantly influence hair follicle morphogenesis, growth, and hair coat traits by modulating genes related to cell proliferation, apoptosis, and differentiation. The biogenesis and activity of these miRNAs are tightly regulated by post-transcriptional processes, epigenetic factors, and feedback mechanisms, ensuring appropriate miRNA expression during development. miRNAs fine-tune critical signaling pathways, such as Wnt, Notch, and TGF-*β*, to regulate hair follicle cycling, immune responses, and inflammation, ultimately shaping the Cashmere Goat’s hair characteristics (79, 86). miRNAs influence hair follicle development by regulating key signaling pathways through target genes. Overexpression of miRNA-29a/b1 inhibits HFSC differentiation by targeting LRP6, ctnnb1, and Bmpr1a, affecting the Wnt and BMP pathways, and leading to shortened hair cycles and hair loss (89). TGF-*β*1 negatively regulates hair follicle growth, but miR-122-5p carried by adipose stem cells (ADSCs) can promote hair growth by inhibiting the TGF-β pathway. FGF is another key pathway, with FGF10 enhancing hair follicle development (89). In cashmere goats, miR-184 was found to promote secondary hair follicle proliferation and inhibit apoptosis, influencing cashmere growth (79, 90). Lastly, the PI3K/Akt pathway is essential for hair follicle regeneration, facilitating epidermal-dermal signal transmission, aiding hair follicle regeneration (91).

### 3.2 Cell-type specific functions of miRNAs

Highly expressed miRNAs typically play key roles in regulating specific cell and tissue types. For instance, miRNA-205 is predominantly found in skin progenitor and stem cells, particularly in hair follicle stem cells (HFSCs) located in the bulge. Studies show that miRNA-205 regulates the PI3K pathway by directly targeting PI3K regulatory genes, influencing hair follicle cell development (92). Furthermore, miR-184 interacts with fibroblast growth factor 10 to promote cell proliferation and inhibit apoptosis in dermal papilla cells of Cashmere goat secondary hair follicles. Chi-miR-370-3p, crucial for fetal hair follicle morphogenesis in Inner Mongolia cashmere goats, inhibits epithelial cell and dermal fibroblast proliferation by targeting FGFR2 and TGF-*β*R2, enhancing migration without promoting apoptosis (93). Overexpressed chi-miR-877-3p promotes hair follicle cell proliferation by inhibiting IGFBP5 expression. miRNA-214, however, inhibits HFSC proliferation and differentiation, altering the cell cycle by targeting enhancer of zeste homologous protein 2, disrupting Wnt/β-catenin signaling. miRNA-124 also aids HFSC differentiation by targeting Sox9. miRNA-203 regulates hair follicle development in cashmere goats by targeting DDOST and NAE1 (83, 93).

In summary, miRNAs target related genes and regulate cell growth during hair follicle development. However, most studies are *in vitro*, with fewer *in vivo* verifications. Further studies are needed to explore the impact of these results in complex body environments (83). The regulation of miRNA biogenesis and activity in velvet goat hair follicles is subject to complex mechanisms (94). These include post-transcriptional control involving RNA-binding proteins, epigenetic regulation like DNA methylation, and feedback loops that ensure spatiotemporal expression of miRNAs (95). These multi-layered regulatory processes fine-tune miRNA expression, allowing precise control over gene expression during hair follicle development. miRNA can regulate gene expression post-transcriptionally by combining the 5′ end sequence of the miRNA and the 3′ non-coding sequence (UNT) of the homologous mRNA sequence, and can regulate gene expression through base complementation (96). Pairing recognizes mRNA and inhibits the translation of target mRNA according to the degree of complementarity. miRNA-203 can specifically bind to the 3’UTR of DDOST and NAE1, resulting in less expression of the target genes DDOST and NAE1 in the telogen (14). The expression level of miRNA-1-3p in the growth phase of cashmere follicle cycle is lower than that in the telogen phase (83). FGF14 is a member of the fibroblast growth factor family. miRNA-1-3p can specifically bind to the 3′UTR of FGF14 mRNA, resulting in FGF14 The expression level is down-regulated, which in turn causes the development of hair follicle cells to transition from the anagen phase to the catagen phase (97). miR-let7a is expressed in cashmere goat skin tissue, and its expression level is high during the early stages of hair growth. It can regulate the hair follicle cycle of Liaoning cashmere goats by acting on *C-myc, FGF5* and *IGF-1R* genes (98).

**Figure 2:**
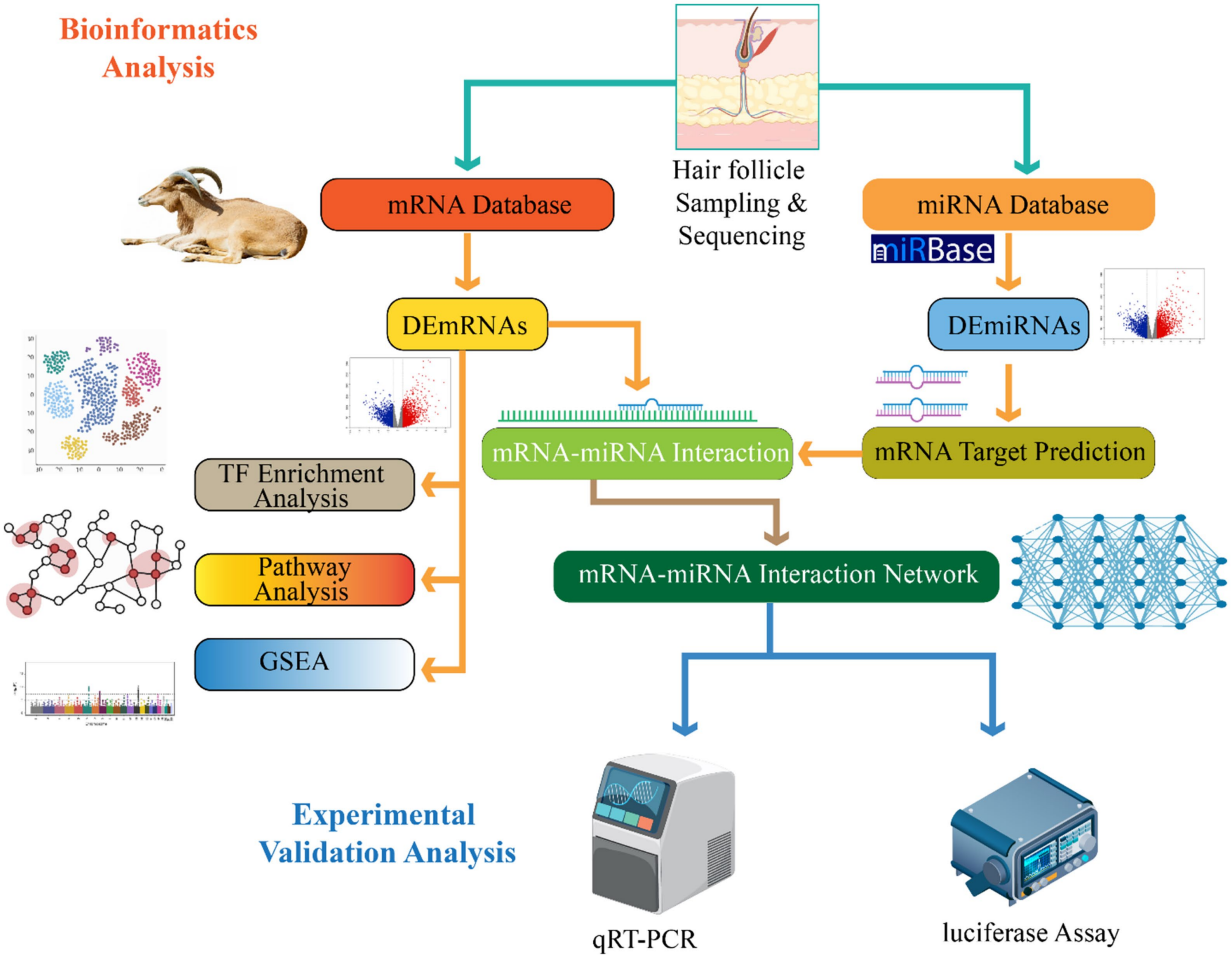
This demonstrates pipeline for identifying mRNA–miRNA interactions involved in hair follicle development. Hair follicle samples undergo sequencing and data mining through mRNA and miRNA databases to identify differentially expressed mRNAs (DEmRNAs) and miRNAs (DEmiRNAs). Subsequent analyses include transcription factor enrichment, pathway analysis, gene set enrichment (GSEA), target prediction, and construction of an mRNA–miRNA interaction network. Predicted interactions are validated experimentally using qRT-PCR and luciferase assays.

The expression of chi-miR-877-3p is down-regulated in the telogen phase, while the expression of the IGFBP5 gene is up-regulated. This is because the overexpression of chi-miR-877-3p in dermal cells inhibits the expression of IGFBP5 gene and promotes cell growth proliferation (82). The TGF-*β*R1 gene belongs to a member of the TGF-β family and has multiple functions such as regulating cell proliferation, differentiation, and apoptosis (99). Studies have shown that miRNA-1298-5p can communicate with the 3′ end of the TGF-βR1 gene (100). Sequence-specific complementary binding of the coding region negatively regulates its expression, thereby regulating the growth and development cycle of Inner Mongolia Cashmere goat villi (21). In summary, miRNA affects the hair follicle development cycle of cashmere goats by specifically binding to the 3’UTR of its target gene to up-regulate or down-regulate the expression of its target gene. These research results provide new insights into the study of hair follicle development cycle (21, 101) (Figure 2).

### 3.3 miRNAs in hair shaft formation, coat color, and hair fineness

MicroRNAs (miRNAs) play a pivotal regulatory role in hair shaft formation, coat color determination, and hair fineness in goats. These small non-coding RNAs regulate gene expression post-transcriptionally by binding to complementary sequences in the 3′ untranslated regions (UTRs) of target mRNAs, leading to mRNA degradation or translational repression (102). Additionally, miRNAs can also interact with the 5’ UTR, coding sequences, and even gene promoters, demonstrating a broad range of regulatory mechanisms (103). In some cases, miRNAs have been found to activate gene expression, further highlighting their versatility. Their subcellular localization also influences their regulatory function, as miRNAs can move between cellular compartments to modulate both translation and transcription rates (104). In the context of coat color formation, miRNAs have emerged as critical regulators by modulating the expression of pigmentation-related genes. For example, miR-129-5p influences melanin production by targeting key genes such as TYR and TYRP1, thus playing a significant role in skin and coat pigmentation. miR-200a contributes to pigmentation regulation by targeting WNT5A and FZD4, which show differential expression patterns among goats with various coat colors (105). Additional miRNAs like miR-27a, miR-193b, and miR-125b-5p further refine pigmentation pathways. miR-27a downregulates WNT3A and KITLG, miR-193b enhances the expression of WNT10A and GNAI2, while miR-125b-5p inhibits melanin synthesis by targeting MITF (106).

Beyond pigmentation, miRNAs are also involved in defining hair length and fineness. Overexpression of miR-29a/b1 has been associated with shorter hair by inhibiting the differentiation of hair follicle stem cells (HFSCs) and suppressing critical components of the Wnt and BMP signaling pathways (79). In cashmere goats, particularly the Liaoning breed, miRNAs such as miR-93 and miR-17-5p influence cashmere fiber fineness, partly by regulating the NFKBIA gene. Beyond pigmentation, miRNAs are also involved in defining hair length and fineness (107). Overexpression of miR-29a/b1 has been associated with shorter hair by inhibiting the differentiation of hair follicle stem cells (HFSCs) and suppressing critical components of the Wnt and BMP signaling pathways (108). In cashmere goats, particularly the Liaoning breed, miRNAs such as miR-93 and miR-17-5p influence cashmere fiber fineness, partly by regulating the NFKBIA gene. In velvet goats, the role of miRNAs extends deeply into hair follicle development and morphogenesis (107). During the early stages of hair follicle formation, specific miRNAs control the proliferation and differentiation of stem and progenitor cells by targeting transcription factors and signaling molecules essential for hair follicle initiation and patterning (109). As follicles mature, miRNAs continue to modulate gene expression in components of the hair shaft, inner and outer root sheaths, and the dermal papilla—structures crucial for proper hair formation and function. Furthermore, miRNA expression in velvet goat hair follicles is highly tissue specific (110). Certain miRNAs are preferentially expressed in different compartments of the follicle, such as the dermal papilla, outer and inner root sheaths, and hair follicle stem cells (111). This spatial specificity enables precise regulation of gene expression necessary for the proper development, differentiation, and cycling of hair follicle cells. Dysregulation of these miRNAs has been linked to abnormalities in hair coat characteristics, reinforcing their essential role in maintaining hair follicle integrity and function (106, 107).

## 4 Molecular mechanisms of miRNA action

### 4.1 Transcriptional regulation

In many mammals, hair follicle (-IF) development follows a highly regulated and periodic cycle encompassing distinct morphological phases: anagen, catagen, and telogen. These dynamic changes are orchestrated through complex molecular networks involving various classes of RNA molecules, including long non-coding RNAs (IncRNAs), messenger RNAs (mRNAs), and micro as (miRNAs) (82). Studies across multiple species—including mice, American mink, rabbits, yaks, and goats—have underscored the evolutionary conservation and species-specific aspects of hair follicle regulation (15, 60). Hair follicles originate from stem cells and are surrounded by dermal fibroblasts. They undergo rapid, cyclic phases of growth, regression, and rest, driven by tightly coordinated gene expression programs (112). Recent transcriptomic analyses using RNA-sequencing technology have revealed that during different stages of sheep HF development specifically at two fetal stages (E90d and El 20d) and at birth, many IncRNAs (461), mRNAs (1,009), and miRNAs (106) exhibit differential expression patterns (113). These expression changes suggest a strong regulatory role of non-coding RNAs in transcriptional and post-transcriptional modulation of HF growth (114). The expression dynamics of these RNA molecules reflect their potential interactions and regulatory roles. Certain IncRNAs, such as Inc-000133 and IncRNA-HOTAIR, have been implicated in HF development, though their precise mechanisms remain incompletely defined (115). Among mRNAs, key players such as FZD4, FZD9, DKKI, and SAM have shown significant differential expression during HF formation in fine wool sheep, indicating their possible involvement in Wnt signaling pathways known to control follicular morphogenesis (116). Moreover, several miRNAs with known or putative roles in follicle regulation were identified. For instance, miR-143 has been associated with HF development in sheep, while members of the miR-200 family are known to influence cell adhesion and morphogenesis within hair germ cells (18, 116). In this context, miR-200b may contribute specifically to HF growth in Aohan fine wool sheep (AFWS). Likewise, miR-21 and miR-143 demonstrated significant expression variation, supporting their involvement in the transcriptional regulation of target genes associated with HF development (114).

Functional analyses of miRNA-IncRNA and miRNA-rnRNA networks provided further insights into gene regulatory mechanisms. Notable regulatory axes such as miR-200a-MSTRG.313133, and miR-21/miR-143 targeting IncRNA MSTRG.172759, emerged as potential transcriptional regulators during distinct stages of follicle growth (117). The involvement of these miRNAs in cell adhesion, signal transduction, and morphogenesis highlights their critical role in modulating the transcription of genes integral to follicular development (118). Additionally, other regulatory pairs such as miR-150-KRTI and miR-370-3p-KRT32/KRT35/VCAN/FOXN1 further emphasize the role of miRNAs in directing HF-related gene expression (118, 119). For example, miR-150 is implicated in HF-related melanoma, whereas miR-370-3p is linked to proliferative pathways (83). Genes such as KRT32 and KR735 are associated with hair structure and morphology, while VCAN serves as a marker for hair papilla cells and is reportedly unaffected by environmental light intensity (120). The transcription factor FOXN7 may play a role in the growth of secondary follicles, indicating that these miRNA-rnRNA interactions modulate key transcriptional regulators involved in skin and hair biology (121).

**Figure 3:**
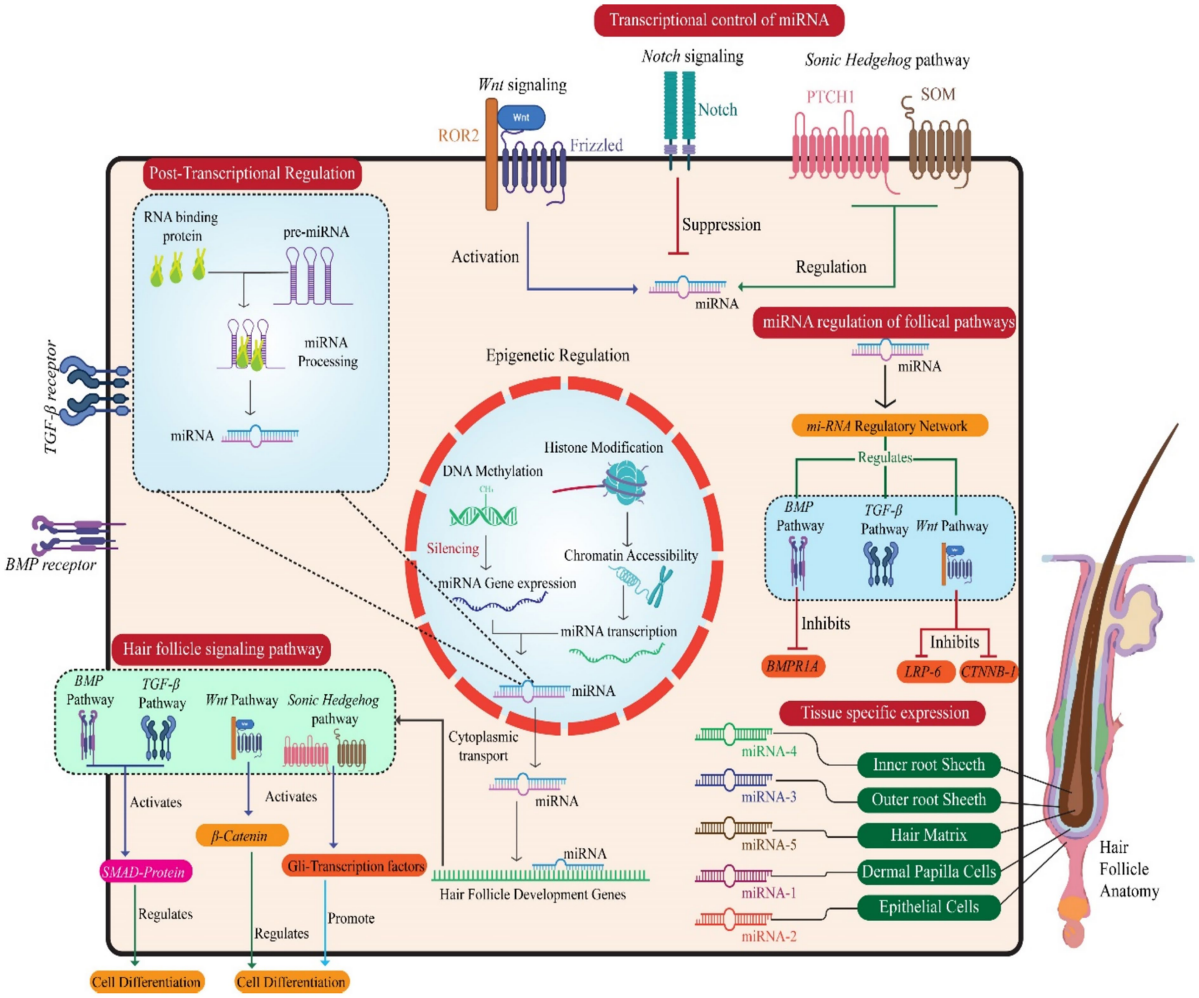
Illustration of miRNAs’ multilayered regulation of hair follicle development, encompassing transcriptional, post-transcriptional, and epigenetic mechanisms. Key follicular signaling pathways (Wnt, Sonic Hedgehog, Notch, BMP) are modulated by miRNAs, affecting pathway components and transcription factors. miRNAs influence hair follicle-specific cell types and regulate genes critical for follicle morphogenesis and cell differentiation. The central nucleus panel depicts miRNA gene expression control, while peripheral interactions highlight how miRNA networks regulate cellular behavior and tissue specificity in hair follicle formation.

In velvet goat hair follicle cells, miRNAs also subject to transcriptional control. This regulation fine-tunes their expression patterns and functions (64). Transcription factors, epigenetic modifications, and chromatin remodeling complexes collectively manage the expression of miRNA genes, ensuring that miRNA levels align with the developmental and functional demands of hair follicles (96). For instance, transcription factors associated with hair follicle morphogenesis—such as those from the Wnt, Notch, and Sonic Hedgehog signaling pathways—directly regulate specific miRNAs in velvet goat hair follicle cells. This precise control is crucial for proper hair follicle growth and development (122). Collectively, these findings elucidate the multifaceted roles of miRNAs and their interaction networks in regulating transcription during hair follicle development. By influencing coding and non-coding targets, miRNAs contribute significantly to the molecular orchestration of follicular growth and differentiation (123) (Figure 3).

### 4.2 Post-transcriptional and epigenetic modifications

In velvet goat hair follicle cells, miRNAs are regulated not only at the transcriptional level but also through post-transcriptional mechanisms, which profoundly affect their biogenesis and functions (124). For instance, RNA-binding proteins modulate the processing of miRNA precursors, and mature miRNAs are selectively exported and compartmentalized within subcellular structures like the nucleus, cytoplasm, and exosomes, influencing their activity (125). These post-transcriptional processes allow precise control of miRNA expression and functionality, enabling hair follicle cells to adapt quickly to developmental signals and maintain cellular balance (125). Ultimately, the regulation of miRNAs in velvet goat hair follicles results from a complex interaction of transcriptional, post-transcriptional, and signaling pathway-mediated processes (96). In velvet goat hair follicle cells, miRNA expression is regulated not only through transcriptional and post-transcriptional mechanisms but also via epigenetic control (89).

This study elucidates the pivotal role of miR-22 as a post-transcriptional regulator involved in hair follicle involution by negatively modulating keratinocyte progenitor expansion, differentiation, and hair shaft assembly during the catagen and telogen phases (56). Through gain- and loss-of-function analyses, miR-22 is shown to promote the anagen-to-catagen transition, prolong the telogen phase, and inhibit re-entry into anagen, establishing its essential function in terminating the active growth phase of the hair cycle (40). They found that miR-22 executes this role by enhancing apoptosis and repressing master transcriptional regulators critical for keratinocyte differentiation. During anagen, transient amplifying matrix cells undergo rapid proliferation and differentiation to form the hair shaft, which is composed primarily of type I and II keratins (126). These keratins generate cytoplasmic intermediate filaments that contribute to cytoskeletal integrity. As hair follicles transition from anagen to catagen and telogen, there is a physiological regression characterized by follicular degeneration and shortening (44). Transcriptomic profiling during these phases shows progressive downregulation of keratin genes, emphasizing the requirement for suppressing keratinocyte differentiation and hair shaft formation for effective follicle involution (45, 56).

miR-22 is identified as a central molecular regulator that orchestrates this process by targeting transcription factors such as DIx3, Msx2, Hoxe13, Foxnl, Nfe213, 615, Smad6, Left DIx4, and MITF (127, 128). Direct interactions were confirmed through the identification of miR-22 binding sites in the 3′ untranslated regions of Nfe2l3, DIx3, Hoxe13, and Foxnl, with luciferase reporter assays validating the post-transcriptional repression of DIx3, Hoxe13, and Foxnl (129). These transcription factors are integral to activating hair-specific keratin genes such as K32, K35, and K37 (130). The inverse expression pattern of miR-22 and DIx3 across hair cycle stages, alongside the phenotypic similarities between DIx3 knockouts and miR-22 overexpressing models, supports a model in which miR-22 downregulates DIx3 to facilitate hair follicle regression (131). Similarly, Foxnt, another direct target, is known to regulate keratin gene expression and thymic epithelial differentiation. The impaired thymic development observed in miR-22 overexpressing mice further substantiates Foxnts role as a functionally relevant target (131, 132). Moreover, the downregulation of 58 keratin genes in miR-22 overexpressing models reinforces its regulatory function in suppressing keratinocyte differentiation (133). This role aligns with the effects seen upon global deletion of the microRNA processing enzymes Dicerl and Drosha, where failure of catagen entry and impaired follicular degradation are observed, further supporting the involvement of miRNAs in follicle regression (56). The less severe phenotype of miR-22 knockout mice compared to Dicerl or Drosha mutants suggests the contribution of additional microRNAs in orchestrating hair cycle transitions. miR-22 shares expression dynamic with other microRNAs such as miR-24 and miR-125b, which also exhibit upregulation during catagen and peak expression in telogen (134). These miRNAs likewise suppress keratinocyte differentiation and promote follicular regression by targeting transcriptional regulators like TCF3 and the vitamin D receptor, respectively (135, 136). Several additional microRNAs—rniR-29a, miR-27a, miR-27b, miR-30a, miR-152, and miR-143—also mirror the expression profile of miR-22, suggesting a combinatorial regulatory network that governs hair cycle termination and maintenance of quiescence (131). In addition to modulating differentiation, miR-22 impairs hair follicle stem cell (HFSC) colony formation and hair neogenesis, minoring the suppressive effects of miR-24 and miR-125b on HFSC self-renewal and lineage commitment (137). This implies a broader role for these miRNAs in modulating both HFSC dynamic and downstream differentiation programs. The catagen phase is known to be driven by apoptosis, and miR-22 further contributes to this process by repressing transcripts of anti-apoptotic regulators including ADAMTS20, Foxc1, GCLC, MITF, SGK3, Msx2, and Vhnl, several of which are directly targeted via miR-22 binding (136, 137). These findings suggest that miR-22 triggers apoptotic pathways to facilitate follicular regression, representing a post-transcriptional mechanism regulating programmed cell death in hair cyding. Finally, emerging evidence links miR-22 expression to androgen signaling, particularly testosterone-induced upregulation (138). Given that androgens are implicated in the premature anagen-to-catagen transition characteristic of androgenic alopecia, this raises the possibility that miR-22 may contribute to the pathophysiology of male pattern baldness. This hypothesis integrates post-transcriptional gene regulation, apoptosis, and hormonal control within the framework of hair follicle biology (56).

Epigenetic modifications, such as DNA methylation and histone modifications, can impact the accessibility of miRNA gene promoters, affecting their transcriptional activity (139). For instance, DNA methylation and histone deacetylation have been shown to suppress the expression of specific miRNAs, leading to disruptions in signaling pathways and developmental processes (140). On the other hand, histone acetylation and other mechanisms that promote open chromatin enhance the transcription of miRNAs, facilitating normal hair follicle development and function (141).

### 4.3 Complex regulatory loops in hair follicle biology

The hair follicle (1-IF) represents a highly dynamic biological system that undergoes cyclic phases of growth, regression, and quiescence. These phases are each characterized by distinct patterns of gene activation and silencing, which are essential for the regulation of HF function (142). MicroRNAs (miRNAs) play a pivotal role in gene silencing mechanisms, and their involvement in regulating hair cycle-related tissue remodeling provides significant insight into the control of hair growth and epithelial tissue renewal (143). By understanding how miRNAs modulate gene expression during the hair cycle, we can further elucidate the complex regulatory networks that govern HF biology (144).

A comprehensive miRNA expression profiling in the skin has revealed substantial alterations in miRNA expression at various stages of the murine hair cycle, offering important insights into the molecular dynamics underlying hair follicle activity (123). Notably, miR-31 was found to exhibit a marked upregulation during the anagen phase, followed by a significant downregulation during catagen and telogen (44). When antisense miR-31 inhibitors were applied to mouse skin during the early and mid-anagen phases, accelerated hair growth was observed, accompanied by altered differentiation of hair matrix keratinocytes and modified hair shaft formation (45). This suggests that miR-31 serves as a critical modulator of HF growth and differentiation. Further molecular analyses, including microarray, qRT-PCR, and Western blot techniques, demonstrated that miR-31 exerts a repressive effect on key growth factors and signaling molecules, including Fgf10, Sclerostin, BAMBI, and Dlx3, as well as keratin genes (145). A luciferase reporter assay confirmed that miR-31 directly targets Krt16, Krt17, 01×3, and Fgf10, underscoring the crucial role of miR-31 in regulating the molecular networks that drive HF growth and involution (56).

In addition to miR-31, miR-214 has been identified as a potent inhibitor of the Writ signaling pathway within keratinocytes. MiR-214 exhibits mutually exclusive expression patterns with (80). By reducing the expression of p-catenin and other downstream components of the Wnt pathway, such as c-myc, cyclin D1, and Pten, miR-214 effectively suppresses Wnt signaling activity. Luciferase reporter assays further confirmed that 8-catenin is a direct target of miR-214 (146). These findings suggest that miR-214 plays a significant role in regulating skin development and regeneration, particularly by modulating 0- catenin levels and controlling Wnt signaling dynamics in keratinocytes. Furthermore, the investigation of miRNAs in the context of BMP signaling has revealed a novel mechanism through which miRNAs mediate the effects of BMP pathways in the skin (147). Treatment of primary mouse keratinocytes with BMP4 resulted in distinct changes in the expression of several miRNAs, including miR-21, which was notably downregulated in response to BMP4 exposure (148). Conversely, miR-21 expression was significantly elevated in the skin of transgenic mice overexpressing the BMP antagonist Noggin, indicating a regulatory feedback loop between miR-21 and BMP signaling (149). Functional analysis through miR-21 mimic transfection demonstrated that miR-21 differentially regulates two groups of 8MP target genes, suggesting that miR-21 acts as a critical modulator of BMP signaling in keratinocytes (81). This highlights an additional layer of complexity in the regulatory circuits that govern HF biology, where miRNAs are positioned as integral players in mediating signaling pathway crosstalk and maintaining the balance of gene expression during skin homeostasis (150).

Overall, miRNAs represent key regulators of gene expression in the skin, especially during the hair cycle. Their ability to target a wide array of growth regulatory molecules, transcription factors, and cytoskeletal proteins underscores their pivotal role in the fine-tuned regulation of HF biology (36). By modulating these molecular pathways, miRNAs ensure the establishment of an optimal gene expression balance within keratinocytes, which is crucial for the maintenance of HF function and skin homeostasis (151). The complex regulatory loops involving miRNAs and their interaction with signaling pathways such as Wnt and BMP add a sophisticated layer of control to the dynamic process of hair follicle development, growth, and regeneration (20).

## 5 Computational, multi-omics, and AI-based approaches

### 5.1 Single-cell RNA-seq and spatial transcriptomics in hair follicle research

Wool, a vital textile raw material, is synthesized by the hair follicle of sheep. Understanding the molecular mechanisms that regulate hair follicle development is therefore crucial for improving wool quality and production (152). Despite its importance, the cellular heterogeneity and limited molecular and spatial characterization of sheep hair follicles have hindered the full elucidation of these mechanisms. Consequently, the genetic and molecular basis of hair follicle development and wool curvature in sheep remains insufficiently understood (153). To address this gap, single-cell RNA sequencing (scRNA-seq) was employed to dissect the cellular composition of lambskin tissues with curly and straight wool phenotypes (154). Single-cell suspensions were prepared from these tissues and subjected to unbiased scRNA-seq analysis. Dimensionality reduction using UMAP revealed 19 distinct cell populations from a total of 15,830 single-cell transcriptomes (155). Each cell population was characterized based on specific gene egression profiles. Novel marker genes were also utilized to identify dermal papilla (DP) cells isolated *in vitro*, enhancing the understanding of cellular identity and lineage (156). To further investigate cellular differentiation, pseudo time trajectory analysis was conducted. This approach enabled the construction of a differentiation pathway for matrix progenitor cells, which commit to the formation of the hair shaft and inner root sheath (IRS) (155). Dynamic gene expression patterns along this trajectory provided insights into the regulatory events guiding hair follicle maturation. Intercellular communication between mesenchymal and epithelial compartments was inferred using CellChat and ligand-receptor interaction data (24). This analysis highlighted robust signaling activity and critical pathways facilitating crosstalk between cell types. Notably, signaling pathways involving Wnt/I3-catenin, fibroblast growth factor (FGF), and bone morphogenetic protein (BMP) were prominently involved, underscoring their regulatory role in hair follicle development (155). Differential gene expression analysis was performed in specific regions of interest, particularly the dermal papilla (DP) and hair mesenchyme (HM) (157). Using the EdgeR package, DEGs were identified by comparing these regions to control compartments (158). To ensure specificity, genes associated with keratin expression—attributable to epithelial contamination—as well as ribosomal and histone genes were excluded from the analysis (159). In the DR(Dermal Region) genes such as RSPO4, RSPO3, BMP4, DKK3, CRABP1, APCDD1, NRG2, and WIF1 were significantly upregulated. These genes are known to participate in signaling cascades important for follicular development (160). In contrast, the HM showed enrichment of genes including KRT85, KR1-35, LEF1, MSX1, and HOXC13, which are indicative of differentiation toward the hair matrix (155).

Validation of these findings was achieved through *in situ* RNA hybridization (RNA-ISH), confirming the localized expression of MSX1 and HOXC13 in the HM, and BMP4, RSPO4, DKK3” (Dickkopf-related protein 3)., and LGR6 in the DP (161). LGR6, the receptor for R-spondins, is commonly associated with stem cell populations and was notably observed in the lower isthmus region of the follicle (162). To further explore molecular interactions essential for follicle development, receptor-ligand interaction analysis was conducted using a curated receptor-ligand database and the NicheNet algorithm (163). Key interaction pairs included RSPO4-LGR6, RSPO3-LGR6, BMP4-ACVR1, BMP4-BMPR2, FGF10-FGFR1, and WNT5A-FZD7, primarily within the DP and HM (164). These findings reinforce the involvement of R-spondin and BMP-mediated signaling, which play crucial roles in follicular differentiation and hair growth. Gene Ontology (GO) and Gene Set Enrichment Analysis (GSEA) further validated the enrichment of Wnt signaling pathways within the hair follicle microenvironment (165). Collectively, these results provide a comprehensive and unbiased view of the cellular landscape, lineage trajectories, spatial signatures, and intercellular communication networks involved in sheep hair follicle development. They also offer valuable molecular insights into the mechanisms underlying wool curvature, paving the way for targeted sheep breeding strategies and enhanced wool traits (155).

### 5.2 Integrating transcriptomics, proteomics, and Epigenomics: a multi-omics approach

Hair follicles (1-IFs) are intricate mini-organs characterized by continuous self-renewal in mammal’s post-birth (166). They consist of dermal and epidermal cell lineages, which orchestrate the dynamic processes of hair growth. Recent studies have identified various genes involved in hair follicle growth and development, such as Wnt10a, Lef1, Sox9, and BMP4 (167). Furthermore, signaling pathways like BMP, Eda, Shh, and TGF-B are known to regulate the growth and development of hair follicles, either promoting or suppressing this process (168). The advancements in genomics have led to a growing focus on environment–gene interactions, with epistatic regulation serving as a potential bridge between the environment and genetic factors. Research on the rearing of cashmere goats under controlled lighting conditions has shown a significant increasein cashmere production during non-cashmere seasons (169). It was proposed that these goats have acquired epigenetic memory, continuing this production pattern in subsequent years even after the experiment ceased. Wu et al. further noted a significant increase in melatonin-related “Hoxc13 expression, which positively regulates genes associated with hair follicle development (170). Given that melatonin levels fluctuate seasonally, it is understood that environmental factors may influence the genetic factors governing hair follicle development. The potential roles of miRNAs in Hu sheep lambskin with different wool patterns were explored by identifying 37 differentially expressed miRNAs (DE-miRNAs) in hair follicles between the small waves (SM) and straight wool (ST) groups using RNA-seq (18, 171). Key miRNAs, such as oar-miR-143, oar-miR-200b, oar-miR-10a, oar-miR-181a, oar-miR-10b, and oar-miR-125b, along with miRNA-mRNA interactions (e.g., miR-125b targeting CD34, miR-181a targeting FGF12 and LMO3, miR-200b targeting ZNF536), were identified (18). These miRNAs and their associated mRNAs may indirectly or directly affect hair follicle development, potentially influencing wool curvature. This discovery offers valuable insight into the molecular mechanisms underpinning wool pattern formation (18).

The interaction between dermal papilla cells (DPCs) and hair matrix cells (HMCs) in yak was further analyzed to examine the molecular basis of hair follicle development (155). Differential mRNA and miRNA expression were characterized, revealing significant differences between DPCs and HMCs (172). Functional enrichment analysis indicated that highly expressed genes in DPCs were associated with hair follicle development pathways, while those in HMCs were enriched in microbiota and immunity-related pathways (173). A total of 39 marker genes for DPCs were identified using 10x genomics single-cell transcriptome data. Additionally, 123 relatively specific miRNAs were screened, including those involved in hair follicle development, such as miR-143, miR-214, miR-125b, miR-31, and miR-200. These findings suggest a complex molecular interaction between DPCs and HMCs during hair follicle development in yak (174). To further explore the molecular mechanisms involved in hair follicle development, small RNA and mRNA libraries from the fetal skin of cashmere goats were constructed at 45, 55, 65, and 75 days, followed by sequencing using Illumina Hiseg4000 (96, 175). Expression profiles of miRNAs and mRNAs in these samples were obtained, identifying differentially expressed miRNAs and mRNAs in six control groups. qRT-PCR experiments confirmed the accuracy of the sequencing results. Sixty-six miRNAs related to secondary hair follicle development were identified, and 33 target genes of these miRNAs were predicted using TargetScan and miRNA (96, 176). A total of 664 mRNAs associated with secondary hair follicle development were screened and GO enrichment and KEGG pathway analyses revealed that some miRNA target genes were consistent with mRNAs involved in secondary hair follicle development (96). Notably, these genes were enriched in the Notch and TGF-I3 signaling pathways. Regulatory networks, such as miR-145-5p, miR-27b-3p, miR-30e-5p, miR-193b-3p-TGF-I31, miR-181b-5p-NOTCH2, and miR-103-3p-NOTCH2, were constructed. Dual-luciferase reporter assays confirmed a targeted relationship between chi-miR-30e-Sp and DLL4, providing a molecular basis for miRNA-mRNA interactions in hair follicle development (96).

In a separate study involving 18 Merino sheep, miRNA data was analyzed from skin tissues at four embryonic stages (E65, E85, E105, El35) and two postnatal stages (D7 and D30). A total of 87 differentially expressed miRNAs (DE-miRNAs) were identified. Heatmap clustering analysis divided these stages into two major developmental phases. An analysis of DE-mRNAs and DE-miRNAs in Stage A revealed that nine DE-miRNAs and 17 DE-mRNAs exhibited targeting relationships. Notably, miR-23b and miR-133 were found to target and regulate ACVR1 B and WNT10A. In dermal fibroblasts, overexpression of miR-133 reduced the mRNA and protein expression of ACVR18, while miR-23b overexpression similarly reduced WNT10A expression (116, 177). This comprehensive study provides new insights into the molecular foundations of hair follicle development, laying the groundwork for improved breeding strategies in fine-wool sheep. The identification of miRNAs and their target genes related to hair follicle development offers a theoretical basis for molecular breeding approaches aimed at enhancing wool production (177).

### 5.3 AI-driven biomarker discovery and therapeutic target prediction

Hair loss and scalp disorders, affecting nearly 80 million individuals in the United States, result from a variety of factors, including aging, stress, medication use, and genetic predisposition (178). Diagnosing these conditions can be particularly challenging, as many early-stage symptoms often go unnoticed, leading to delays in appropriate treatment (179). In particular, distinguishing between normal hair shedding and pathological hair loss requires professional dermatological evaluation, which can be time-consuming and contribute to worsening conditions (179, 180). In recent years, neural network-based image-processing technologies have proven valuable in healthcare, particularly in the early detection of severe diseases such as cancers and tumors. These applications assist clinicians in diagnosing complex conditions by providing insights into early-stage pathologies, thus facilitating faster intervention (181). In this study, we explore the use of deep learning techniques to predict three prevalent types of hair loss and scalp-related diseases: alopecia, psoriasis, and folliculitis. Despite the challenges posed by a limited number of studies, scarce dataset availability, and significant variability in publicly available images, we were able to compile a dataset of 150 scalp images sourced from multiple repositories (182). The images underwent extensive preprocessing steps—including denoising, image equalization, enhancement, and data balancing to reduce the error rate and enhance model performance (183). A 2D convolutional neural network (CNN) was trained on this processed dataset, resulting in an overall training accuracy of 96.2% and a validation accuracy of 91.1%. The model’s performance was further evaluated using precision and recall metrics for each condition: alopecia, psoriasis, and folliculitis, which yielded scores of 0.895, 0.846, and 1.0, respectively (178). Furthermore, we developed and curated a comprehensive scalp image dataset for future research, enabling further advancements in Al-driven biomarker discovery and therapeutic target prediction for scalp-related diseases.

**Figure 4:**
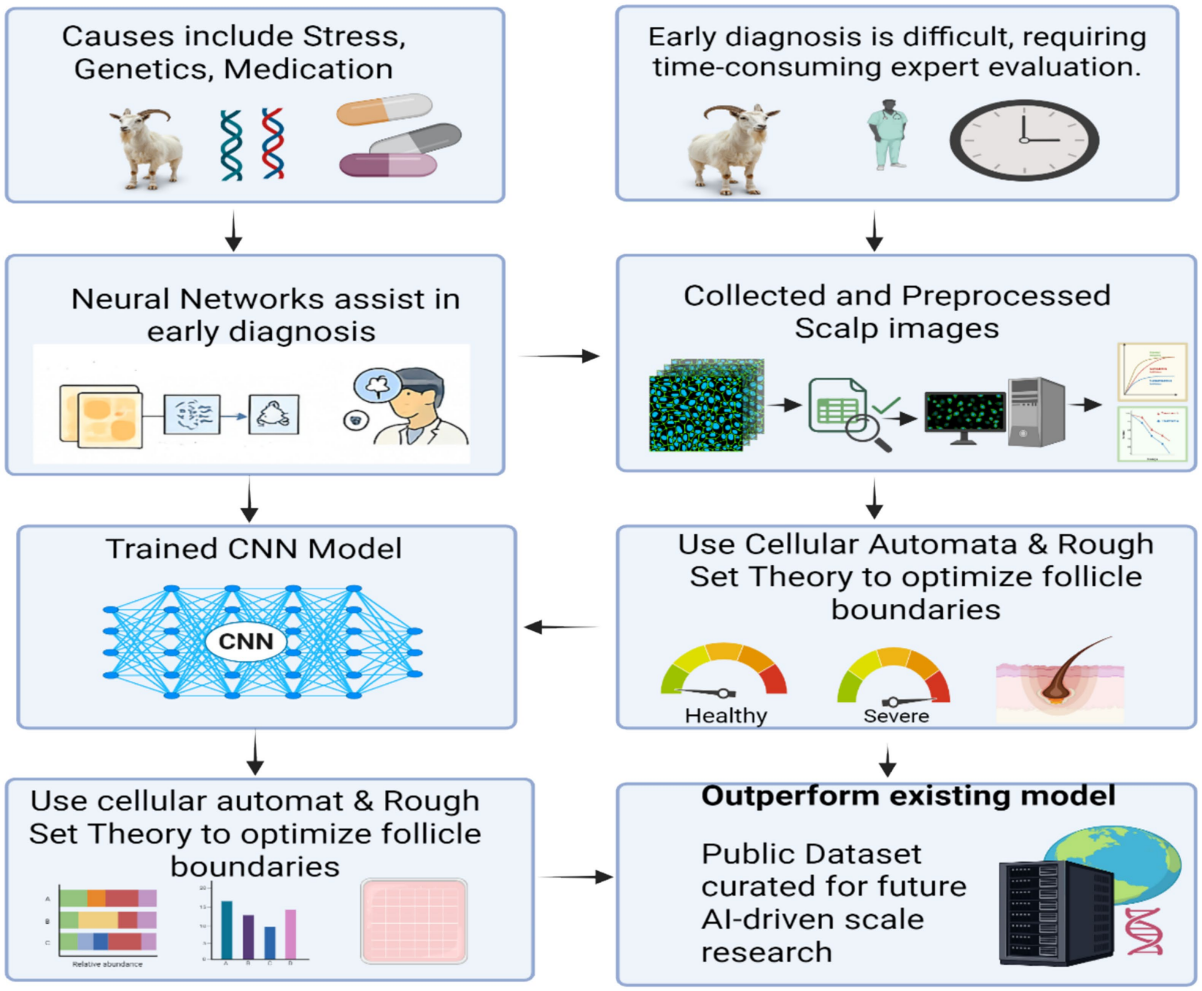
This flowchart presents an AI-assisted approach to diagnosing hair follicle conditions, addressing challenges such as causes (stress, genetics, medication) and the need for expert evaluation. Scalp images are collected, preprocessed, and analyzed using neural networks and trained convolutional neural networks (CNNs). Cellular automata and rough set theory are applied to optimize follicle boundary detection. The resulting model outperforms existing methods and generates a public dataset to support future AI-based dermatological research.

Deep learning techniques have demonstrated significant potential in automating the process of hair loss detection, yet challenges remain in ensuring high precision and reliability, particularly when it comes to determining the severity of hair loss (184). To address these challenges, a novel framework the Cat Swarm Optimization-based Convolutional Neural System (CS-CNS), has been proposed for hair follicle segmentation and status classification. The CS-CNS approach begins with the collection of hair follicle images, which are subsequently trained within the system (185, 186). The collected dataset undergoes preprocessing using the Adaptive Wiener Filter (AWF) to reduce noise and enhance image quality. Feature extraction is then performed using the Hexagonal Scale Invariant Feature Transform (H-SIFT), which ensures robust feature representation, even under scale and rotation variations (187). To further improve the accuracy of segmentation, Cellular Automation based Rough Set Theory (CA-RST) is employed (188). This technique optimizes the segmentation process by refining the boundaries of hair follicles, ensuring more precise identification and classification (184). In the classification phase, the fitness of the cat swami is updated to enhance the accuracy of hair follicle status prediction, distinguishing between normal, severe, and healthy conditions. Each hair follicle is assigned a score, which is computed and adjusted to fall within a defined range of 0 to 2 (189). Finally, the performance of the CS-CNS model is evaluated against existing methods using key metrics such as accuracy, precision, recall, F1-score, and error rate. These experimental results highlight the effectiveness of the proposed model in comparison to other prevailing systems, demonstrating its potential for reliable and precise hair follicle status classification (190) (Figure 4).

## 6 Translational and therapeutic applications

### 6.1 miRNA-based therapeutics

MicroRNAs (miRNAs) are emerging as crucial regulators in the development of hair follicles, playing key roles in various stages of hair growth, including hair regeneration, hair cycle progression, and androgenetic alopecia (AGA) (191, 192). Notably, miRNAs can modulate important signaling pathways, such as the WM/8-catenin pathway, which is integral to hair follicle development. Understanding how specific miRNAs influence this pathway is essential for designing effective miRNA-based therapeutics. One miRNA of interest is miR-218-5p, which has been shown to promote hair regeneration in mice by targeting SFRP2 (193). This suggests that miR-218-5p plays a pivotal role in modulating hair follicle activity. However, studies on this miRNA have not focused on its impact on DKK1 levels, a key modulator of Wnt/8- catenin signaling. The Wnt/8-catenin pathway is known to be involved in numerous pathophysiological processes, including cancer, and miRNAs can influence this pathway by targeting proteins such as DKK1 (194). The regulation of this pathway by miRNAs is thus critical for advancing hair follicle regeneration strategies, particularly in the context of AGA. A well-studied miRNA in AGA is ssssssssmiR-125, which is abundantly expressed in balding dermal papilla (DP) cells. miR-125 inhibits the vitamin D receptor (VDR), which is essential for hair growth through its activation of Wnt/8-catenin signaling (195). The downregulation of VDR by miR-125 impairs hair growth, emphasizing its potential role in the pathophysiology of AGA. Another miRNA, miR-126, is present in hair follicles and may be involved in regulating hair growth, though further research is needed to fully understand its role (17, 192).

In contrast, miR-133b has been found to have a negative impact on hair growth. Its levels are significantly elevated in patients with AGA, and this upregulation correlates with a reduction in p-catenin levels in hair dermal papilla cells (196, 197). These findings suggest that miR-133b acts as a suppressor of hair growth, possibly by inhibiting Wnt/p-catenin signaling. Moreover, the expression of miR-133b, along with miR-141-5p, miR-652- 5p, and miR-1247-5p, has been observed to be unregulated in AGA-affected regions, further highlighting their potential involvement in the condition (198). The versatility of miRNAs is also reflected in their ability to regulate multiple genes and signaling pathways. miR-29a is a prime example, as it inhibits DKK1, KRM2, and SFRP2, all of which are antagonists of Wnt/p-catenin signaling. The inhibition of these proteins by miR-29a enhances Wnt/p-catenin activation, thus promoting hair growth (194). In diabetic mice, increased levels of miR-29a correlate with elevated 3-catenin levels and reduced DKK1 expression, reinforcing its role in modulating the Wnt/p-catenin pathway and suggesting its potential therapeutic value for hair regeneration in conditions like AGA (199). miR-31 also plays a significant role in hair follicle development, as it is highly expressed during the anagen phase of the hair cycle. This miRNA targets a variety of genes, including the androgen receptor (AR), which is crucial for the pathogenesis of AGA Elevated levels of miR-31 inhibit AR expression, potentially providing a means of ameliorating AGA symptoms (200). Moreover, studies on miR-31 in hairless mutant mice have shown a downregulation of miR-31, indicating its essential function in hair follicle development (200).

Other miRNAs, such as miR-103 and miR-107, have also been implicated in the regulation of Wnt/p-catenin signaling. These miRNAs target DKK1 and NON2, both of which act as negative regulators of this pathway (194). By downregulating these inhibitory proteins, miR-103 and miR-107 may help activate Wnt/p-catenin signaling, promoting hair growth. Additionally, these miRNAs have been shown to enhance stem-like features in cancer cells, further supporting their potential to stimulate hair follicle stem cells in the context of AGA (194). miR-203, a highly abundant miRNA in the epidermis, has been shown to target DKK1 in various tissues, including lung adenocarcinoma and mesenchymal stem cells. Given its expression in hair follicles, miR-203 may also play a role in promoting hair growth by inhibiting DKK1, thereby activating Wnt/p-catenin signaling (201). These findings highlight miR-203 as a promising candidate for therapies aimed at hair regeneration. In conclusion, miRNAs exert significant control over hair follicle development and regeneration, particularly through the modulation of the Wnt/p-catenin signaling pathway (194). Identifying the key miRNAs involved in this process is critical for developing effective miRNA-based therapeutics for AGA. However, further research is required to fully elucidate the complex interactions between miRNAs and their targets, enabling the design of precise and sustainable treatments for hair loss disorders in cashmere goats.

### 6.2 Applications in precision breeding of cashmere goats

MicroRNAs (miRNAs) have emerged as key molecular regulators with significant implications in precision breeding strategies aimed at improving cashmere traits in goats (202). Cashmere fibers, derived from secondary hair follicles, are renowned for their fine diameter, softness, and luxurious texture, making them highly valuable compared to other animal fibers such as wool, mohair, and yak hair (8). Enhancing the yield and quality of cashmere fibers requires an in-depth understanding of the molecular mechanisms controlling follicular development and fiber characteristics. In this context, miRNAs play a pivotal role in regulating gene expression at the post-transcriptional level, influencing various biological processes including hair follicle development, pigmentation, cell proliferation, and differentiation (202). Numerous miRNAs have been identified to influence the morphogenesis and cyclic activity of hair follicles. For instance, miR-31, miR-22, and miR-214 are involved in follicular morphogenesis, apoptosis, and keratinocyte proliferation, respectively, while miR-205 and miR-125b are key regulators of hair follicle stem cell activity (13). Comparative miRNA expression profiling in caprine skin tissues of different goat breeds has revealed breed-specific differences in miRNA abundance, correlating with variations in fiber yield and quality (200). Notably, miR-26a-5p, one of the most abundantly expressed miRNAs in caprine skin, is known to promote dermal papilla cell proliferation and enhance follicular morphogenesis through the TGF-I3/SMAD signaling pathway (203). Similarly, miR-199a-5p enhances hair follicle development by directly modulating the Wnt signaling pathway, which is essential for hair follicle formation and regeneration (204).

Mother important miRNA, miR-143-3p, has been implicated in increasing the number of secondary hair follicles, potentially contributing to higher cashmere fiber yield (148). In contrast, miR-486-5p was significantly downregulated in high-yielding goats, potentially reducing its inhibitory effects on growth-promoting genes such as VEGFA and BCL118, which are involved in hair follicle enlargement and density. These findings suggest that suppression of specific miRNAs may enhance follicular activity and fiber production (59). Furthermore, miRNAs also influence cashmere fiber pigmentation. Downregulation of miR-129-5p in white-fiber-producing goats corresponds with its known inhibitory role in melanin biosynthesis via suppression of TYR and MITE, indicating its utility in selecting for fiber color (84). Similarly, novel miRNAs such as novel-m0002-5p, targeting Sox10 and MITF, highlight additional regulatory axes that modulate melanocyte function and pigmentation traits. Among the newly identified miRNAs, several novel miRNAs were found to directly target genes associated with cashmere fiber structure and follicular function (205). For example, novel-m0147-3p targets KRTAP24-1, a gene encoding keratin-associated proteins that influence fiber diameter, while novel-m0152-3p targets MSI2, which maintains hair follicle stem cell quiescence and may suppress fiber growth (206). These novel regulatory elements offer potential targets for genetic manipulation to optimize fiber diameter and yield. Functional annotation of miRNA target genes revealed significant enrichment in pathways related to binding activity, cellular protein modification, and signaling pathways such as Writ, MAPK, and Notch (207). The Wnt signaling pathway is central to initiating and maintaining hair follicle development and regeneration by activating hair follicle stern cells. The MAPK pathway, in addition to regulating follicle development, also influences melanin synthesis through phosphorylation of MIFF, thereby linking it to fiber coloration (208). The Notch signaling pathway contributes to follicular growth by regulating cellular differentiation and maintaining the developmental integrity of hair follicle (209). In summary, miRNAs represent powerful molecular tools in precision breeding strategies for cashmere goats. Their regulatory roles in hair follicle development, fiber quality, and pigmentation provide a framework for selecting genetic markers and potential targets for gene editing to enhance desirable cashmere traits. Integrating miRNA-based insights into breeding programs holds promise for improving fiber yield, fineness, and color in a breed specific and targeted manner.

**Figure 5:**
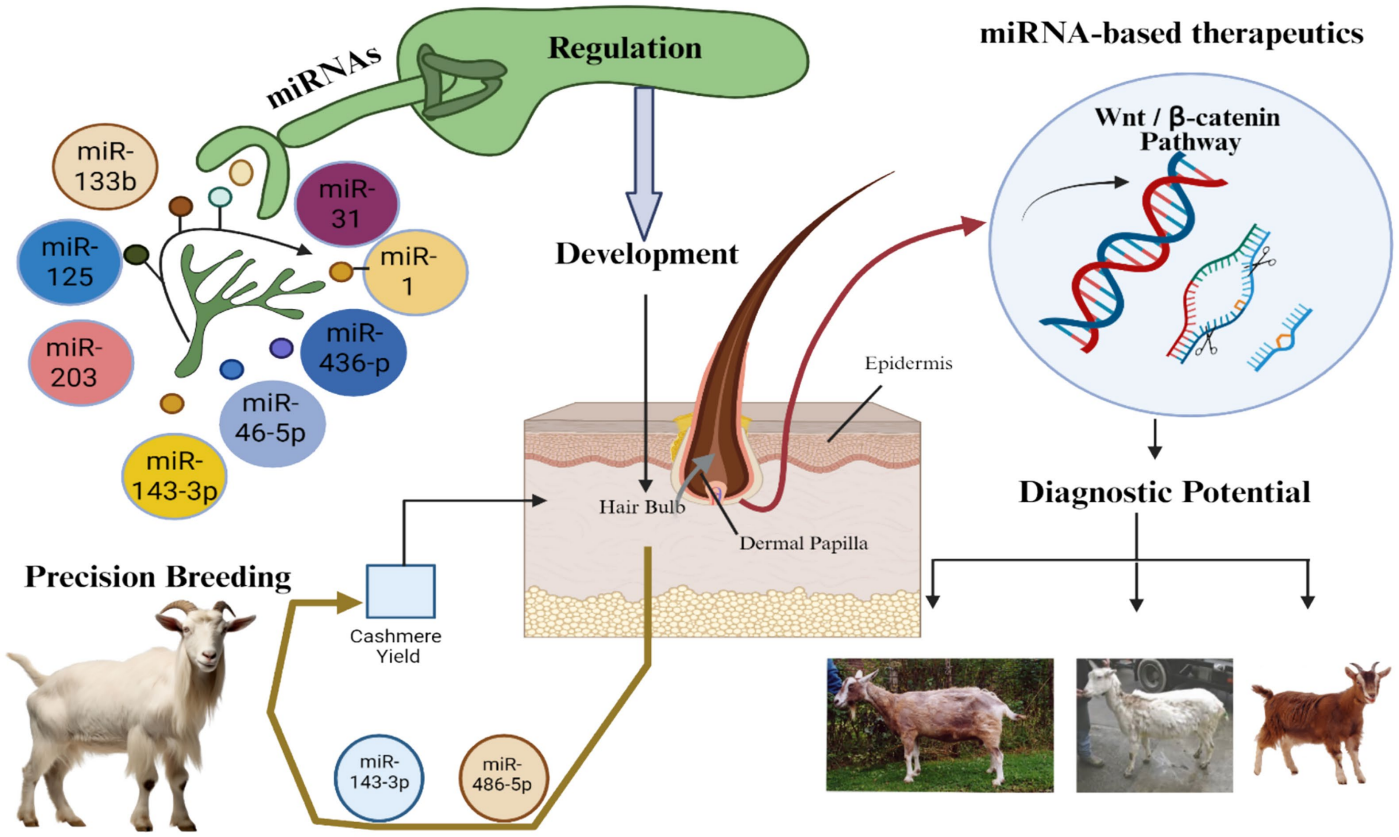
MicroRNA-mediated regulation of hair follicle development and Wnt/β-catenin signaling in cashmere goats. Central nodes depict key miRNAs (e.g., miR-125b, miR-31, miR-203, miR-143-3p, miR-133b) and their interactions with target structures such as the dermal papilla and Wnt/β-catenin pathway. Arrows indicate regulatory effects on hair bulb activity, follicular regeneration, and pathway modulation. Peripheral images illustrate cashmere goats with varying fiber characteristics, reflecting miRNA influence on yield, density, and pigmentation. This regulatory network highlights candidate targets for both therapeutic intervention and precision breeding.

In Figure 5, several core miRNAs are depicted for their regulatory influence on hair follicle development via the Wnt/*β*-catenin pathway. miR-125b, highly expressed in balding dermal papilla cells, represses vitamin D receptor (VDR) expression, thereby downregulating Wnt pathway activation and impairing hair growth (202). miR-143-3p, conversely, promotes secondary follicle density and dermal papilla cell proliferation, contributing to increased cashmere fiber yield (210). miR-203 is shown inhibiting DKK1, a known antagonist of Wnt signaling, thereby indirectly sustaining Wnt/β-catenin activation and promoting follicular regeneration (211). Other miRNAs like miR-133b and miR-31 exhibit dual roles: miR-133b acts as a suppressor by reducing β-catenin levels, while miR-31 is active during anagen and promotes keratinocyte proliferation and follicle elongation (212). miR-486-5p, found to be downregulated in high-yielding goats, is implicated in inhibiting pro-growth genes such as VEGFA and BCL11B, potentially acting as a brake on follicle expansion. These interconnected nodes emphasize the polygenic and phase-specific regulatory network by which miRNAs shape hair follicle morphogenesis, density, and phenotype in both clinical and agricultural contexts.

### 6.3 Diagnostic potential of miRNA signatures

MicroRNAs (miRNAs) have gained considerable attention in recent years for their crucial roles as post-transcriptional regulators of gene expression involved in autoimmune responses (213). Notably, various studies have identified their dysregulation as being intricately linked to autoimmune-associated hair loss disorders, such as Alopecia areata (AA), androgenetic alopecia (AGA), and frontal fibrosing alopecia (FFA), highlighting their potential as diagnostic biomarkers (214). Alopecia areata (AA), an autoimmune disorder characterized by patchy hair loss with a global prevalence of approximately 0.1%, has been closely associated with aberrant miRNA expression patterns in skin tissues (215). In AA, autoreactive T lymphocytes infiltrate the peribulbar region of anagen hair follicles, disrupting their immune privilege and triggering follicular damage. Comparative microarray analyses using C3H/HeJ mice, which spontaneously develop AA, revealed differential expression of nearly 100 miRNAs relative to healthy controls (216). These miRNAs are implicated in key immunological pathways, including JAK–STAT signaling, antigen presentation, apoptosis, and interferon signaling (217). Upregulated miRNAs such as mmu-miR-31, mmu-miR-155, and mmu-miR-329, alongside downregulated ones like mmu-miR-100, mmu-miR-1, mmu-miR-26b, mmu-miR-29c, mmu-miR-30b, mmu-miR-101a, mmu-miR-133a, mmu-miR-365, mmu-miR-451, and mmu-miR-705, were identified as potentially critical contributors to M pathogenesis (216).

Complementary to these findings, human studies have also provided evidence supporting the diagnostic relevance of miRNAs in AA (3). A microarray analysis using blood samples from severe AA patients uncovered 36 significantly differentially expressed miRNAs, comprising 22 upregulated and 14 downregulated miRNAs when compared with healthy individuals (218). Among these, miR-125b-5p and miR-186-5p were found to synergistically modulate inflammatory responses and tissue injury, while miR-185-5p was implicated in broader inflammatory mechanisms (219). In particular, miR-186-5p targets the transcription factor FOXO1, suggesting its involvement in modulating cell metabolism, cycle progression, and apoptosis—processes that may drive AA progression (220). The role of miRNAs extends beyond AA and into other hair loss conditions, such as androgenetic alopecia (AGA). In a comparative microarray analysis of scalp tissue from AGA patients and healthy controls, 43 differentially expressed miRNAs (21 upregulated, 22 downregulated) were identified (221). Among the upregulated candidates, hsa-miR-1247-5p, hsa-miR-652-5p, hsa-miR-520d-5p, hsa-miR-141-5p, and hsa-miR-133b were validated through qRT-PCR. These miRNAs are postulated to target genes involved in cell signaling, hormonal regulation, and cell cycle processes critical to hair follicle physiology, thus implicating them in the molecular underpinnings of AGA (222). Similarly, in frontal fibrosing alopecia (FFA), a disease marked by progressive frontotemporal hairline recession, circulating miRNAs in plasma samples have shown promise as diagnostic indicators (223). Analysis using a Human Fibrosis miRNA PCR Array revealed that hsa-let-7d-5p, hsa-miR-18a-Sp, hsa-miR-19a-3p, and hsa-miR-20a-Sp exhibit disease-state-specific expression patterns. These miRNAs co-regulate overlapping sets of gene targets, reinforcing their functional relevance (224).

Table 1 summarizes key microRNAs involved in hair follicle development, cycling, and pigmentation in velvet goats. Highlighted miRNAs such as miR-203, miR-214, and miR-29a/b1 regulate crucial pathways like WNT, BMP, and TGF-*β*, influencing follicle morphogenesis and hair quality. Others, like miR-129-5p and miR-200a, affect pigmentation by targeting melanin-related genes. Several miRNAs also show diagnostic or therapeutic potential, offering insight for biomarker development and precision breeding. Notably, the miRNA-7 family, among the most evolutionary conserved and abundantly expressed miRNAs in the epidermis, is believed to orchestrate key cutaneous differentiation processes (225). Taken together, these findings underscore the diagnostic potential of miRNA signatures in autoimmune hair loss disorders (226). The consistent differential expression of specific miRNAs across various conditions and tissue types not only reflects their contribution to disease pathophysiology but also highlights their utility as minimally invasive biomarkers for early detection, disease monitoring, and potentially targeted therapeutic interventions (227). Given the potential of noncoding RNAs in regulating human hair-related disorders, they may also be applicable for treating hair follicle-associated diseases in cashmere goats and sheep.

**Table 1:** Key microRNAs involved in hair follicle development.

miRNA	Function/Role	Functional Category	Target Genes/Pathways	Diagnostic Potential	Reference
miR-203	Regulates epidermis and hair follicle development	Hair Cycle Regulation	DDOST, NAE1	High (biomarker for telogen phase)	(211)
miR-200b	Hair follicle development via WNT pathway	Hair Follicle Morphogenesis	WNT pathway	Not reported	(228)
miR-125b	Regulates epidermal thickness and prevents fur formation	Epidermal Regulation	Vitamin D receptor	High (linked to alopecia)	(229)
chi-miR-17-5p	Regulates BAMBI and SMAD1	Hair Structure Regulation	BAMBI, SMAD1	Potential (involved in HF regulation)	(21)
chi-miR-199a-5p	Regulates BAMBI and SMAD1	Hair Structure Regulation	BAMBI, SMAD1	Potential	(21)
chi-miR-214-3p	Blocks H19s effect and suppresses DPC proliferation	Cell Proliferation	I^2^-catenin	Moderate (proliferation suppression)	(87)
miRNA-21	Negatively regulates hair follicle genes	Hair Development	CNKSR2, KLF3, TNPO1	Moderate (downregulated in BMP signaling)	(88)
miRNA-29a/b1	Inhibits HFSC differentiation	HFSC Regulation	LRP6, ctnnb1, Bmpr1a	High (linked to hair loss)	(230)
miR-122-5p	Promotes hair growth by inhibiting TGF-Î^2^	Hair Growth Enhancement	TGF-I^2^ pathway	Therapeutic use (via ADSCs)	(231)
miR-184	Promotes SHF proliferation, inhibits apoptosis	Hair Growth	FGF10	High (promotes hair growth)	(232)
miR-205	Regulates HFSCs via PI3K pathway	HFSC Regulation	PI3K	Potential (stem cell regulation)	(20)
chi-miR-370-3p	Inhibits proliferation, enhances migration	HF Morphogenesis	FGFR2, TGF-Î^2^R2	Moderate (inhibits proliferation)	(93)
chi-miR-877-3p	Promotes HF cell proliferation	Proliferation Control	IGFBP5	Potential (proliferation enhancer)	(83)
miRNA-124	Aids HFSC differentiation	HFSC Differentiation	Sox9	Moderate (differentiation modulator)	(233)
miRNA-1-3p	Downregulates FGF14	Transition Phase Modulation	FGF14	Moderate (hair cycle regulation)	(101)
miR-let7a	Regulates hair follicle cycle	Hair Growth Initiation	C-myc, FGF5, IGF-1R	High (regulates cycle genes)	(98)
miRNA-1298-5p	Regulates hair cycle by targeting TGF-Î^2^R1	Growth Cycle Modulation	TGF-Î^2^R1	Potential (cycle regulation)	(234)
miR-129-5p	Affects pigmentation by targeting TYR, TYRP1	Pigmentation Control	TYR, TYRP1	Moderate (pigmentation gene regulator)	(105)
miR-200a	Targets pigmentation genes	Pigmentation Control	WNT5A, FZD4	Moderate (targets coat color genes)	(117)
miR-27a	Regulates pigmentation	Pigmentation Control	WNT3A, KITLG	Moderate (WNT signaling)	(235)
miR-193b	Enhances pigmentation	Pigmentation Enhancement	WNT10A, GNAI2	Moderate (enhancer of pigmentation)	(106)
miR-125b-5p	Inhibits melanin synthesis	Pigmentation Suppression	MITF	High (inhibits melanin synthesis)	(236)

## 7 Critical gaps and future perspectives

The *in vivo* validation of miRNA therapeutics in hair follicle regeneration presents several significant challenges, primarily due to the difficulties associated with effective delivery to target cells while preventing nuclease-mediated degradation. This is a major hurdle in miRNA-based treatments, alongside concerns related to specificity, stability, immune activation, and toxicity in both *in vitro* and *in vivo* environments. For local delivery, naked RNA can be directly injected; however, for systemic delivery, a more sophisticated delivery system is required. RNA molecules and other macromolecules are typically encapsulated in nanoparticles, which are often modified with polyethylene glycol (PEG), cholesterol, or other moieties. Additionally, a specific ligand may be introduced to enhance cellular uptake via the cell membrane. After endocytosis by the cell, the nanoparticle undergoes degradation, and the nucleic acid is released into the cytoplasm. Several delivery systems have been explored for the in vivo validation of miRNA in hair follicle regeneration. Polyethyleneimine (PEI)-based delivery has proven effective, with successful applications in miR-145 and miR-33a delivery in mice models. PEI, a positively charged organic polymer, efficiently forms complexes with anionic RNA, enabling effective transfection. Despite its advantages, PEI, both in its branched and linear forms, presents limitations such as low transfection efficiency and cytotoxicity. Poly Qactide-co-glycolide (PLGA), an FDA-approved biodegradable drug delivery system, has been studied as a carrier for miRNA. However, due to its hydrophobic nature PLGA-based systems exhibit relatively low delivery efficiency. In contrast, poly (amidoamine) dendrimers have demonstrated high transfection efficiency, making them a promising candidate among polymer-based delivery systems for miRNA.

In a specific study on hair regrowth in mice, *in vivo* jet PEl was found to be an effective delivery system for miR-218-5p, offering better safety and efficacy compared to PEI, although it is relatively expensive. Non-viral lipid-based delivery systems, such as lipofectamine, in vivo fectamine, and oligofectamine, are also commonly used in miRNA delivery for hair follicle studies. One promising lipid-based delivery system is the lipid nanoparticle (LNP), which mimics the cell membrane structure and facilitates efficient cellular uptake. LNPs have been used to deliver miR-634, resulting in reduced tumor xenograft growth in mice, and have shown potential in hair follicle regeneration. LNPs are also utilized in mRNA vaccines, such as the COVID-19 vaccine, suggesting their future potential in miRNA-based therapies. However, LNPs face challenges, such as the need for ultra-low-temperature storage, which limits their practical application. Beyond non-viral vectors, viral vectors such as adenoviral, retroviral, lentiviral, and bacteriophage-based virus-like particles have been explored for miRNA delivery. While viral vectors offer high efficiency in gene delivery, they are associated with limitations, such as the low loading capacity of phage vectors and the potential for insertional mutations due to random genomic integration in lentiviral vectors. Exosome-based delivery systems are emerging as a promising non-viral approach, with their ability to efficiently deliver miRNAs. However, the complexity of their preparation remains a significant obstacle to their widespread use. In the context of hair growth studies, lipofectamine is predominantly employed for *in vitro* applications, while *in vivo* jet PEl is used for in vivo delivery of miRNAs. In conclusion, while various delivery systems for miRNA have been identified as effective carriers, challenges related to stability, toxicity, localized delivery, and the integrity of nucleic acids continue to hinder the success in vivo validation of miRNA-based therapies in hair follicle regeneration.

## 8 Conclusion

Hair follicle biology exemplifies a sophisticated system governed by tightly coordinated genetic and epigenetic regulatory mechanisms, with microRNAs playing an indispensable role in fine-tuning gene expression throughout development, cycling, and regeneration. This review consolidates evidence from diverse mammalian models, focusing on cashmere goats, to illuminate the multifaceted functions of miRNAs in controlling hair follicle morphogenesis, stem cell dynamics, and phenotypic traits such as hair length, fineness, and pigmentation. The identification of miRNAs and their target genes within canonical signaling pathways—including WNT/p-catenin, BMP, Notch, and TGF-p—underscores their critical contributions to hair follicle induction, differentiation, and cycling. Cell-type specific miRNAs regulate distinct compartments of the follicle, with emerging data revealing spatially and temporally precise expression patterns that ensure coordinated follicular function. The use of advanced single-cell transcriptomics and spatial mapping has revolutionized our understanding of follicular heterogeneity and intercellular communication, identified novel cell populations and signaling interactions essential for hair growth. Integrating transcriptomic, proteomic, and epigenomic data through multi-omics frameworks has expanded the landscape of hair follicle regulatory networks, uncovering complex miRNA-mediated feedback loops and crosstalk with other non-coding RNAs. The application of artificial intelligence has further enhanced biomarker identification and therapeutic target prediction, particularly in the context of human hair loss disorders such as androgenetic alopecia and autoimmune alopecia. These innovations hold promises for developing personalized medicine approaches and improving livestock breeding efficiency. Therapeutically, miRNA-based interventions targeting critical pathways like WNT/p-catenin demonstrate encouraging results in preclinical models, with the potential to reverse hair loss and promote regeneration. However, effective and safe *in vivo* delivery remains a formidable challenge due to issues of stability, specificity, and immunogenicity. Advances in nanoparticle and viral vector technologies, coupled with targeted ligand conjugation, may overcome these barriers. In agricultural settings, miRNA-guided precision breeding strategies offer a powerful tool for enhancing cashmere quality and yield, addressing both economic and genetic sustainability goals. Nevertheless, significant gaps persist. *In vivo* functional validation of miRNA roles requires more refined delivery systems and animal models that accurately recapitulate human hair follicle biology. Moreover, expanding the understanding of miRNA interactions within the broader non-coding RNA milieu and their epigenetic regulation is critical to fully elucidate follicular gene regulation. Future research must leverage systems biology, integrating high-dimensional data with AI-driven predictive models to decipher these complex networks and translate findings into practical applications. In summary, miRNAs are central regulators of hair follicle biology with profound implications for dermatology and animal science. Continued interdisciplinary efforts combining molecular biology, computational modeling, and translational research will be essential to unlock their full potential for therapeutic innovation and precision breeding ultimately advancing both human health and agriculture productivity.
